# Review of Heavy Metals Pollution in China in Agricultural and Urban Soils

**DOI:** 10.5696/2156-9614-8.18.180607

**Published:** 2018-06-06

**Authors:** Eshetu Shifaw

**Affiliations:** College of Geographical Science, Department of GIS and Cartography, Fujian Normal University, China

**Keywords:** heavy metal, background value, pollution level, spatial distribution, remedial measures

## Abstract

**Background.:**

The concentrations of heavy metals in soil and potential risks to the environment and public health are receiving increased attention in China.

**Objectives.:**

The objective of this paper is to review and analyze heavy metals soil contamination in urban and agricultural areas and on a national scale in China.

**Methods.:**

Initially, data on soil heavy metals concentration levels were gathered from previous studies and narratively analyzed. A further statistical analysis was performed using the geo-accumulation index (I_geo_), Nemerow integrated pollution index (NIPI), mean, standard deviation (SD), skewness and kurtosis. Pollution levels were calculated and tabulated to illustrate overall spatial variations. In addition, pollution sources, remedial measures and impact of soil contamination as well as limitations are addressed.

**Results.:**

The concentration level of heavy metals was above the natural background level in most areas of China. The problem was more prevalent in urban soils than agricultural soils. At the national level, the soil in most of the southern provinces and Beijing were heavily polluted. Even though the pollution condition based on I_geo_ was promising, the Nemerow integrated pollution level was the most worrisome. The soils in about 53% of the provinces were moderately to heavily polluted (NIPI>2). The effects were noticed in terms of both public and ecological health risks. The major sources were waste gas, wastewater, and hazardous residuals from factories and agricultural inputs such as pesticides. Efforts have been made to reduce the concentrations and health risks of heavy metals, including policy interventions, controlling contamination sources, reducing the phytoavailability of heavy metals, selecting and rearing of grain cultivars with low risk of contamination, paddy water and fertilizer management, land use changes, phytoremediation and engineering techniques.

**Conclusions.:**

China is experiencing rapid economic and technological advancements. This increases the risk of heavy metals contamination of soil. If serious attention is not paid to this problem, soil toxicity and biological accumulation will continue to threaten the sustainability of China's development.

**Competing Interests.:**

The authors declare no competing financial interests

## Introduction

Soil is the subsystem for biogeochemical cycles such as nutrient recycling, energy exchange, moderation of greenhouse gas fluxes and recycling of carbon.^1^,^2^ Soil management is key to maintaining high quality food and fiber production for the world's growing population.^3^ However, as agricultural inputs, urbanization and economic development continue, heavy metals are being deposited into soil.^4^,^5^ The concentration of heavy metals in the soil, due to agricultural input, fast urbanization and industrialization is a problem affecting a large area of China.^5^ As heavy metals are not degraded through chemical and physical weathering, their concentrations are increased through time, altering soil properties and minimizing the availability of nutrients for biological activities.^6^

Soil heavy metals pollution degrades soil's inherent capacity for ecological functions. These soil functions include buffering anthropogenic perturbations, sustaining productivity, moderating pollutants, protecting watersheds, improving water and air quality, and others.^2^ There has been significant degradation of these soil functions around the world due to adverse changes in soil's physical, chemical and biological properties and soil protection is an international concern.^7^ Accordingly, soil quality assessment is the main research emphasis of modern soil science for ensuring soil health and sustainability.^1^ Careful consideration is necessary to monitor anthropogenic impacts on soil quality and to closely monitor long-term soil and environmental quality indicators.^8^

Following recognition of soil pollution as a serious problem in China, nationwide surveys of soil were conducted between 2005 and 2013.^9^ These surveys covered more than 70% of China's land area.^10^ The survey reported that 16% of soil samples and 19% of agricultural soil was contaminated with heavy metals (exceeding the environmental quality standard). Of the soils considered to be polluted, 82.4% of contamination was due to metals and metalloids and the rest (17.6%) was due to organic contaminants.^10^ Over 6 million hectares of farmland was polluted with industrial and urban wastes in the early 1990s, and soil affected by acid rain expanded from 1.5 to 2.5 million ha from 1985 to 1994. A relatively recent analysis also reported that about 10 million hectares of arable land in China was polluted by heavy metals.^11^

Even though levels of naturally occurring heavy metals are generally low in China, some studies have reported high levels of pollution of soil, air and water and negative affects on human health.^12^ Protecting food security through soil management is one of the priorities of the central government.^13^ However, the current soil quality monitoring system is insufficient to accurately determine soil contamination status.^13^ Therefore, knowledge about the source, concentration, and pollution level of soil heavy metals is essential to soil remediation and protection.^14^,^15^ This study aims to review the concentration, pollution level, sources and remediation of soil heavy metals in China.

## Methods

First, publications addressing the specific review objectives across different spatial contexts were retrieved. Most of the reviewed papers were based on soil samples at a depth of 10 to 20 cm of topsoil. Sample sizes for the analyses of metals in agricultural soil (*Table 1*) were as follows: 6 (Beijing), 70 (Guangzhou), 76 (Yangzhou), 102 (Wuxi), 60 (Gansu), 30 (Chengdu), 100 (Taihang), 8 (Zhengzhou), 240 (Kunshan), 20 (Xuzhou), 14 (Jinghe), and 26 (Hainan).^16–27^ The corresponding sample size for studies in urban soil (*Table 1*) were 773 (Beijing), 40 (Guangzhou), 273 (Shanghai), 319 (Qingdao), 30 (Jinchang), 82 (Hangzhou), 110 (Changsha), 48 (Hongkong), 215 (Luoyang), 138 (Nanjing), 39 (Changchun), 54 (Taicang), 286 (Fuyang), 20 (Shenyang), 21 (Xuzhou) and 132 (Fuzhou).^28–43^ Soil sample size was generally higher among studies of urban soil than agricultural soil. This may be due to higher levels of metal pollution in urban soil that could not be represented by smaller samples.

**Table 1 —:**
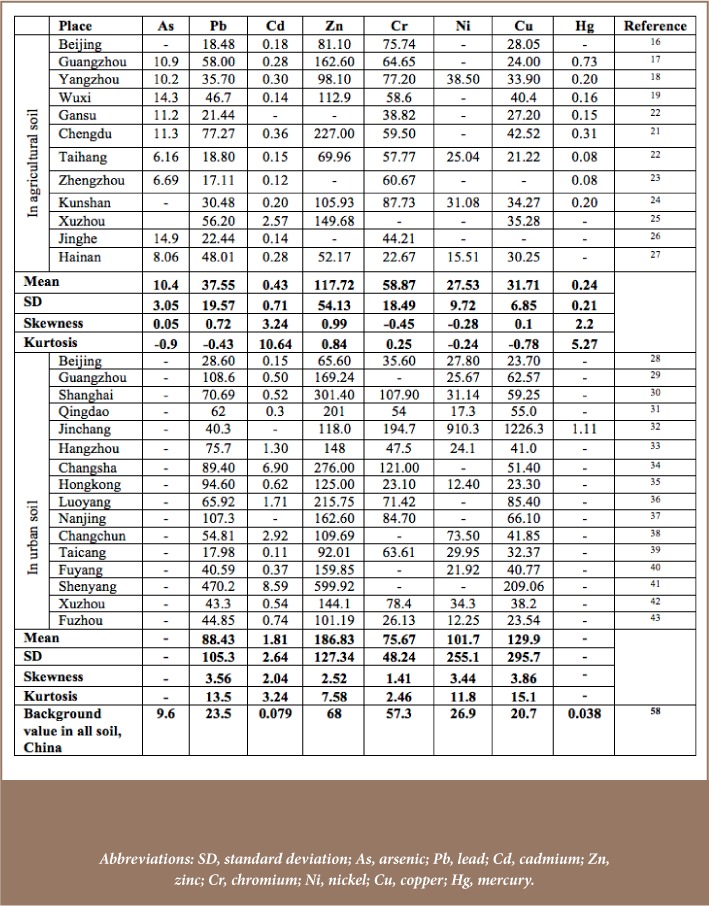
Concentration of Soil Heavy Metals in Agricultural and Urban Soils across Sample Sites (mg.kg)

**Abbreviations**
*I_geo_*: Geo-accumulation index
*NIPI*: Nemerow integrated pollution index
*SD*: Standard deviation

The pollution index is a powerful tool for summarizing soil heavy metal contamination.^44^ Various calculation methods were used such as pollution index (PI) and integrated pollution index, enrichment factor and geo-accumulation index (I_geo_).^45^ Further statistical analysis was performed based on the concentration levels of heavy metals in previous studies. These include the I_geo_, Nemerow integrated pollution index (NIPI), mean, standard deviation (SD), skewness and kurtosis.

### Nemerow integrated pollution index

First, the pollution index and average pollution index (PI_avr_) were calculated.

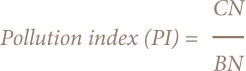
Where Cn is the measured concentration value (mg/kg), and Bn is the soil background values of heavy metals (mg/kg).^46^ It is a single pollution index. The average pollution index is calculated as follows:

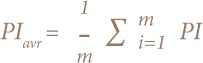
Where PI is the pollution index of heavy metal I and m is the number of heavy metals. After calculating PI and its average, a soil's overall pollution status was derived using the NIPI. This was used to quantify the total pollution status of soil by heavy metals.^47^ It is derived as follows:

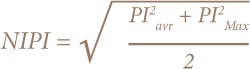
Where PI^2^_avr_ is the average of the PI of all considered metals and PI^2^_max_ is the maximum value. Soil pollution status was classified into five grades based on NIPI: NIPI ≤ 0.7 (safety domain), 0.7 < NIPI ≤1 (precaution domain), 1 < NIPI ≤ 2 (slight pollution), 2 < NIPI ≤ 3 (moderate pollution), and NIPI N ≥3 (heavy pollution).^48^

### Geoaccumulation index

This method was designed in 1969 by Muller.^49^ The formula is given as:

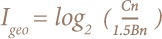
Where Cn is the measured concentration value (mg/kg), and Bn is the soil background value of heavy metals (mg/kg). It deals with the degree of a metals' input due to human activities with regard to environmental geochemistry background values.^50^

To minimize the effects of the variations of each metal's background values in the environment and influence of anthropogenic activities, Muller used the constant 1.5.^44^ He also classified the geo-accumulation index into seven pollution grades: I_geo_ ≤0 (not polluted), 0<I_geo_ ≤1 (slightly to moderately polluted), 1<I_geo_ ≤2 (moderately polluted), 2< I_geo_ ≤3 (moderately to strongly polluted), 3 <I_geo_ ≤4 (strongly polluted), 4<I_geo_≤5 (strongly to extremely polluted) and I_geo_>5 (extremely polluted).^51^

### Soil heavy metals concentrations in China

#### Agricultural soil

Heavy metals concentrations have been rapidly increasing in China. The concentrations of heavy metals were below background values in only a few instances, and all mean concentration values were higher than their reference values (*Table 1*). The greatest variation was observed for zinc (Zn). Non-ferrous metal mining and smelting activities are a major source of Zn and show a high pollution rate (49.04%) in China.^52^ Uneven distribution of mining sites and high Zn production may be responsible for this high spatial variation and elevation over background levels. These results and the variability of other metals are indicators of human influence. High variability indicates greater anthropogenic effects which alter the concentration of heavy metals.^53^ Mercury (Hg) showed the least variability with a SD=0.21, which is very close to the mean (0.24). In addition, cadmium (Cd) showed the highest asymmetric distribution across sites (skewness=3.24 and kurtosis=10.64).

In response to the decline of farmland and the growing demand for food in China, agricultural intensification with modern inputs has increased. Irrigation, and use of chemical fertilizers and insecticides have accelerated the metals concentration of agricultural soils in China.^52^ Chemical fertilizers and insecticides contribute to the high pollution rate for Cd (7.24%) and nickel (Ni) (3.04%), as well as 2% pollution of soil samples by copper (Cu) and Hg. Furthermore, waste discharged from 1.6 million enterprises in townships and others that have recently migrated to rural areas for cheaper land and low labor costs are other major sources of soil contamination. According to a report of the Environmental Monitoring Department in 2009, these enterprises discharged 5.9 billion tons of waste water and 13.2 million tons of particulate emissions in rural areas of China.^54^

#### Urban soil

As seen in Table 1, the concentration levels of heavy metals in urban soil were generally higher than in agricultural soil. This is a clear manifestation of the degree to which anthropogenic factors are contributing to spatial variability of heavy metal concentrations. With the exception of Cr and Ni in some urban areas and Pb in Taicang, the concentrations of all other heavy metals were higher than their background values. The ratio of heavy metals concentrations to background values, known as the pollution index, also differed notably across locations. For instance, Pb (mean 108.6 : background value 23.5) in Guangzhou, Cd (6.90 : 0.079) in Changsha, Zn (599.92 : 68) in Shenyang, as well as Cr (194.7 : 57.3), Ni (910.3 : 101.7) and Cu (1226.3 : 209.1) in Jinchang showed the highest pollution indices, indicating high additions to soil. The higher SDs of some metals reflect differences in the concentrations of heavy metals among study sites.^55^ Additionally, CU experienced the highest asymmetric distribution with a greater degree of peakedness (kkewness=3.86 and kurtosis=15.1). Generally, heavy metals are natural elements of soil and their natural concentrations are low.^56^ However, enhanced concentrations are very harmful and a direct response to urbanization and industrialization.^5^ Unless serious measures are taken, the problem will continue, as urbanization has been rapid in China, increasing from 18% in 1978 to 46.6% in 2009 and is predicted to reach 65% by 2030.^57^

#### Soil heavy metals at the national level

The soil environment has degraded in China due to the continued increase in heavy metals which are toxic to the ecosystem. It is very difficult to recover soil health if soil is polluted with heavy metals.^58^,^59^ The concentration level of heavy metals and their spatial disparities across provinces can be seen in Table 2. Soil metals were greater than their reference levels in most of the provinces. This is especially evident for Cd, Zn and Cr in all provinces. This pollution often goes unnoticed by the public as metal contamination is colorless and odorless.^59^ The concentrations of As and Ni, however, are lower in some provinces (*Table 2*).^60^ Despite spatial variation, mean values indicate the severity of soil metals concentrations and the need for countermeasures. The highest variability in the mean values was observed for Zn (SD=20.01). The peak concentrations of most heavy metals (As, Zn, Ni, Cu and Hg) were found in Guizhou province, indicating that they share the same pollution source. Cadmium concentration distribution showed the highest skew value (skewness=3.25) and highest peakedness (kurtosis=11.47). Despite the increased levels of soil metals, the problem has not received much attention from policy makers in China.^54^ The large number of sites (>1.5 million) and large volume of heavy metals discharges indicates that the trend of increased concentration levels and public and environmental health risk is continuing.^54^

**Table 2 —:**
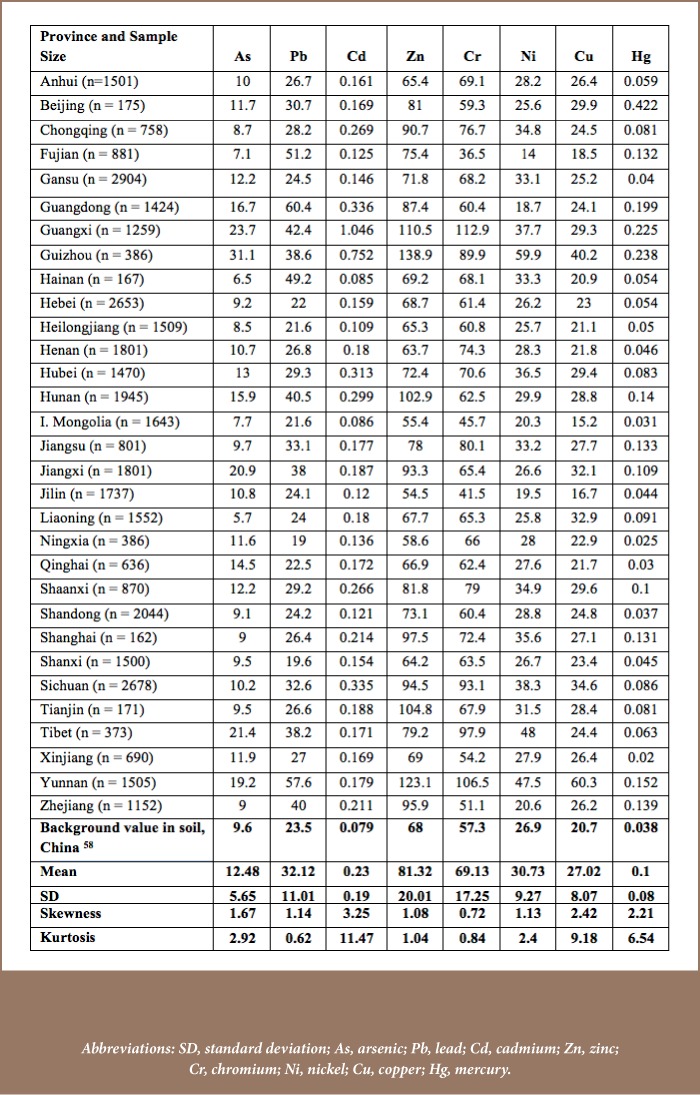
Concentration of Soil Heavy Metals Based on Mean Values (10th Percentile-90th Percentile) Across Provinces in China (mg/kg)

### Pollution level of soil heavy metals

For the analysis of soil heavy metals, I_geo_ and NIPI results are summarized in Table 3. Since its implementation in 1969 to quantify the metal concentration of sediments, I_geo_ has been widely used to identify the pollution level of soil heavy metals.^49^ To evaluate soil environmental quality, different studies commonly use NIPI, as discussed below.^61–63^

**Table 3 —:**
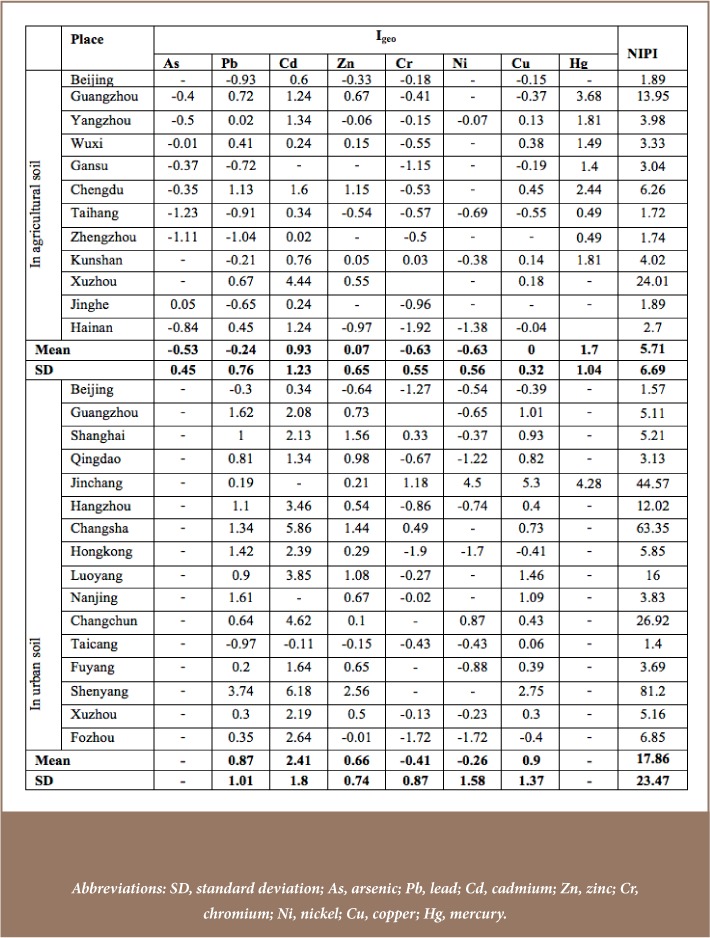
Geo-accumulation Index (I_geo_) and Nemerow Integrated Pollution Index (NIPI) of Heavy Metals in Agricultural and Urban Soil

#### Agricultural soil

Geo-accumulation index values ranged from −1.92 (Cr) to 4.44 (Cd). The mean I_geo_ of As, Pb, Cr, Ni and Cu revealed that agricultural soil was not contaminated by these metals. Cadmium (0.93) and Zn (0.07) were present in soil at light to moderate level contamination levels, whereas Hg (1.7) was moderately contaminated in soil. Heavy metals contamination levels varied across provinces. For example, agricultural soil in Xuzhou was strongly to extremely seriously polluted with Cd (I_geo_=4.44) and strongly polluted in Yangzhou with Hg (I_geo_=3.68). Based on standard deviation, Cd (1.23) and Hg (1.04) showed relatively high variability in their soil pollution levels. Even though the pollution level based on the mean I_geo_ values was promising, total pollution status using NIPI is more troubling. This provides clearer insight into soil environmental quality as this index takes into account both the mean and maximum concentration of heavy metals.^64^ The overall pollution level indicates that agricultural soil is heavily polluted in Xuzhou, Guangzhou, Chengdu, Kunshan, Yangzhou, Wuxi and Gansu provinces (NIPI ≥3). This agricultural soil pollution leads to further pollution of agricultural products. For instance, in Shaoguan, the pollution level of rice is far greater than the maximum permissible limit (0.2 mg/kg) for the average concentration of Cd (0.69 mg/kg) and Pb (0.39 mg/kg), elevating the risk of these metals entering the human body.^54^,^65^

#### Urban soil

The geo-accumulation index (I_geo_) of heavy metals in urban soil is generally higher than in agricultural soils (*Table 3*), indicating that urban soil is relatively more polluted and that the major risk factor is human activity. Spatial disparities in the pollution level of metals were also found to be higher in urban soils than agricultural soils, indicating dissimilar anthropogenic influences and levels of industrialization. Mean I_geo_ values ranged from moderate to strong pollution levels in the case of Cd (2.41) to no pollution for Cr (−0.41). In addition, Cd showed higher I_geo_ values, followed by Pb. The urban soil of most cities was heavily polluted with higher NIPI values (≥3) (*Table 3*). This increasing agricultural to urban gradient of heavy metals soil pollution revealed the corresponding intensity of urbanization and industrialization which enhances environmental damage. This indicates that urban soil quality is deteriorating, with adverse effects on the health of urban green plants, decreased water quality and increased risks to human health.^29^

#### Pollution level of soil heavy metals at the national scale

Heavy metals soil pollution was found in every province. The average I_geo_ value for soil contamination showed slight to moderate pollution for Cd. Only Cd in Guangdon and Sichuan and Hg in Beijing and Gansu showed moderate pollution levels. The overall soil contamination level was evaluated using NIPI, revealing that Beijing, Hubei, Hunan, Guangdong, Guangxi, Sichuan, Guizhou and Yunnan provinces had heavily polluted soils. Inner Mongolia was the only province with a good soil pollution status (0.7 < NIPI ≤1, precaution domain). The rest were in the category of slight to moderate pollution (*Table 4*). It is possible that soil metals pollution has recently increased in China. This is evidenced by the fact that the pollution levels in urban and agricultural soil in Beijing were not found to be elevated in earlier studies as shown in Table 2, while a recent study at the national level found that soil is heavily polluted (*Table 4*). Further enrichment of soil heavy metals in China needs to be remediated by enacting effective measures relying on recent research results.^52^

**Table 4 —:**
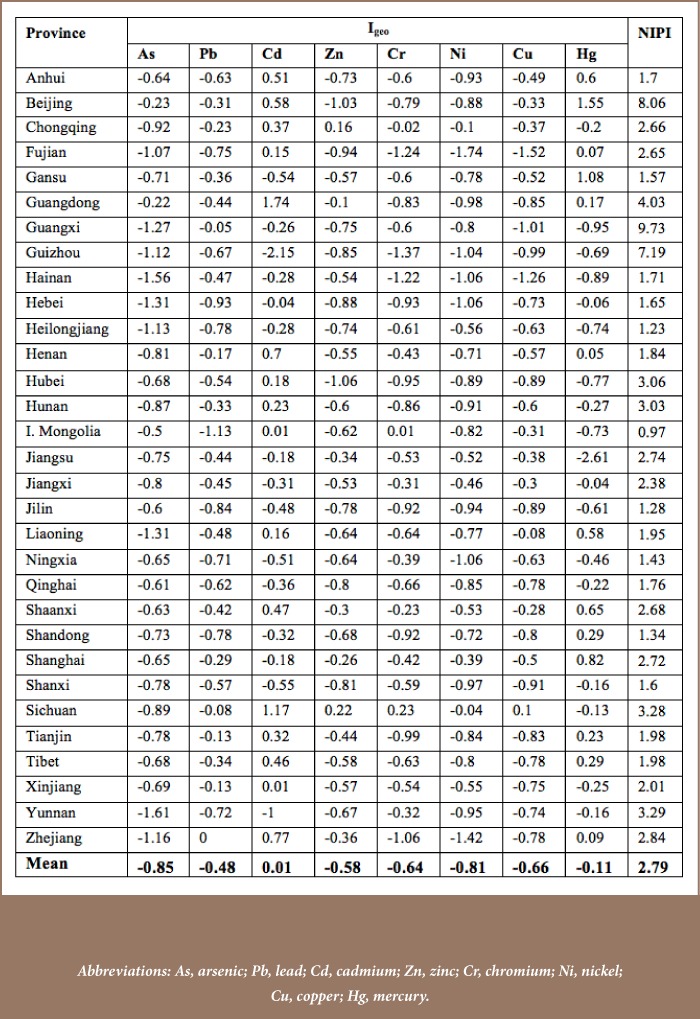
Geo-accumulation Index (I_geo_) and Nemerow Integrated Pollution Index (NIPI) of Heavy Metals Across Provinces in China (mg/kg)

### Sources of soil heavy metals

#### Natural sources

The increase in heavy metals soil pollution is partly attributed to natural factors such as volcanic eruptions and continental dusts, as well as soil's parent materials and pedogenetic processes.^56^,^66^ One of the major reasons for variations in the natural concentration levels of heavy metals in China across different soil types is differences in soil-forming rocks and minerals.^67–70^ The concentrations of several heavy metals were positively correlated with concentrations of iron or aluminium oxides in soils, reflecting the influence of pedogenesis on these elements.^67^,^71^ In addition, the acidic nature of soils is one of the reasons for high accumulations of heavy metals in the tropical and subtropical regions of southern China.^72^ Each soil type has a different capacity to restore soil resilience.^73^ However, the natural level of metals in soil is generally low and does not pose a threat.^56^

#### Anthropogenic sources

Rapid industrialization and urbanization over the last three decades are the main sources of enhanced heavy metals in the environment.^74^,^75^ The dominant sources include sewage irrigation, mining, sludge application and smelting operations for metallic ores, industrial wastes and combustion of fossil fuels.^52^,^76^ Coal consumption in 2010 emitted approximately 9000, 360, 450 and 25000 tons of As, Cd, Hg and Pb, respectively.^72^ Nearly 80% of the total national electricity generation is from coal-fired power plants, which are the most important sources of metal input into soil.^77^ Atmospheric deposition is another source of heavy metal contamination.^78^ The rate of atmospheric deposition in China is higher than in other developed countries. Atmospheric deposition of Cd ranges from 0.4 to 25 g ha^−1^ year^−1^ with a mean of 4 g ha^−1^ yr^−1^ in China.^72^

This figure is substantially higher than the current mean for the European Union (0.35 g ha^−1^ yr^−1^).^79^ Farming practices using water irrigation, animal manure and chemical fertilizers are other contributing factors to increased Cd, Cu and Zn in agricultural soil.^6^ The contribution of different human activities to the metals pollution rate in farmland soil in China is presented in Table 5.^52^

**Table 5 —:**
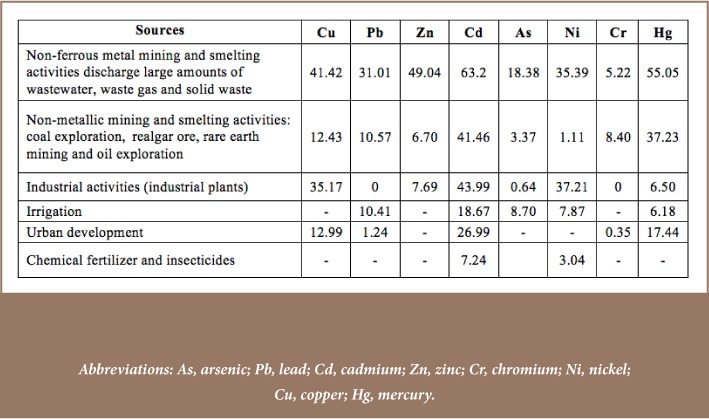
Averages of Heavy Metal Pollution Rates Across Six Anthropogenic Sources (%)

Lack of effective environmental protection policies is another contributing factor for soil pollution. China has a relatively recent legal framework for environmental protection. Protections against soil pollution began in 1972 after the country's participation in Human-Environment interactions at the Stockholm Conference.^80^ Despite China's environmental protection efforts, the rate of soil pollution is not yet under control. Economic and technological development has very often compromised environmental standards.^81^

### Remedial Measures for Heavy metals soil pollution

A comparison of nationwide soil surveys across time shows that concentrations of some heavy metals have significantly increased over the last 25 years.^10^ In addition, contaminated soil sites have been identified and some attempts at remediation have been made over the past 20 years. A comprehensive management project for remediation of contaminated land was launched in 2014 by the Ministry of Agriculture and the Ministry of Finance, and pilot studies were carried out in Changsha, Zhuzhou, and Xiangtan.^13^ An action plan for controlling soil pollution was also approved by the Ministry of Environmental Protection on March 18, 2014. Liability and funding issues were defined in soil pollution policies as shown in Table 6.^82^

**Table 6 —:**
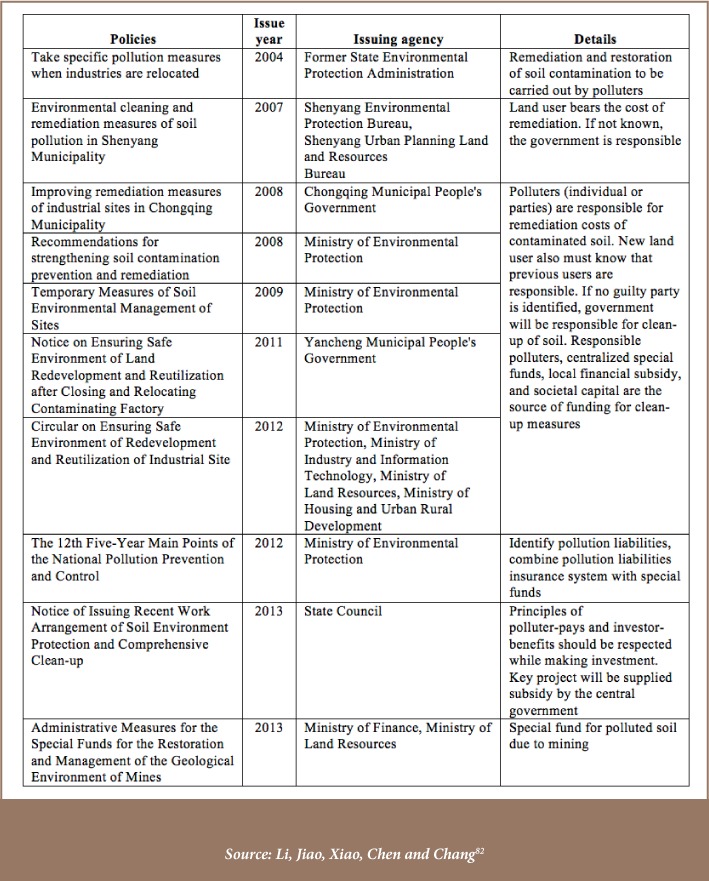
Liability and Funding Issues in Soil Pollution Policies in China

Various remedial strategies are discussed in the following subsections. These strategies are used to minimize heavy metal concentrations in soil or in the edible part of plants to minimize health risks.

#### Controlling sources of contamination

Locating and controlling large pollution emission sources such as mining, smelting and other metal consuming industries is the first step to combatting soil pollution with heavy metals. This requires more rigorous monitoring and enforcement of environmental protection laws.^10^ In 2011, China's first heavy metal pollution control plan for a particular area, Xiangjiang river basin, was officially approved by the State Council.^54^ In the same year, the “12th National 5-Year Plan for Comprehensive Prevention and Control of Heavy Metal Pollution” was approved.^83^ The main goals of this plan are to establish a complete heavy metal pollution control and risk assessment system for the environment and health, effective control of pollution by reducing discharges of major heavy metals (Hg, Cr, As, Cd, and Pb) in key regions (eastern and central China) by 15% between 2011 and 2015, taking 2007 as reference, ensuring that discharges of major heavy metals in non-key regions do not exceed 2007 levels, and significantly reducing pollution incidents.^54^

#### Reducing phytoavailability of heavy metals

An important focus of risk management for polluted soil is the phytoavailability of heavy metals.^10^ The acidic content of soil can be modified to reach the target soil pH (around 6.5) by various materials, but these efforts differ in their capacity for soil acid neutralization, reaction rate and cost. For example, grain Cd and Pb accumulation were decreased by applications of biochar due to its effect on soil pH.^84^,^85^ However, up to 40 t ha^−1^ biochar is required to achieve these reductions, making this a high cost remediation method.^84^,^85^ In addition to biochar, other materials such as sepiolite, sewage sludge, red mud and oilseed rape have an impact on heavy metal immobilization in soil.^86^,^87^

#### Selecting and rearing of grain cultivars with low risk of contamination

The uptake and distribution of heavy metals varies across and within crop species.^88^ Identifying cultivars' genetic variation in heavy metal accumulation is essential for replacing high-accumulating with low-accumulating cultivars.^10^ A number of rice cultivars have been shown to uptake Cd into their root cells and sequester it in the vacuoles.^89^,^90^ There is a rather high initial cost for identifying and rearing such cultivars, with costs declining later when more breeding lines are generated.^88^

#### Paddy water and fertilizer management

One of the major sources of dietary As and Cd is paddy rice, and severity varies across different water management practices. Field experiments in seven major rice cultivars by Hu et al. indicated a significant increase of soil acid (hydrochloric acid) and extractable As concentrations, and a decrease in extractable Cd.^91^ In addition, soil redox status heavily affects the bio-availability of some elements (Cr, Fe, As) and is directly influenced by the amount of soil moisture. This highlights the importance of water management in minimizing heavy metal contamination.^92^ A study of Japanese rice grains suggested that the concentration trend of two important pollutants (As and Cd) was not managed using the water management method alone due to differences in the properties of the soils.^93^ The application of silicone fertilizer has a positive effect in reducing As concentration in rice straw (78%) and rice grain (16%), but negatively affects soil by increasing its contamination.^94^ The application of zinc fertilization with red mud, rape straw and corn straw minimized Cd pollution in both soil and vegetables.^95^ Furthermore, application of organic matter in the form of Chinese vetch has been shown to fix pollutants in soil.^92^ The combined use of organic and inorganic fertilizer in the subtropical region of China has minimized soil pollution by increasing microbial biomass carbon, nitrogen and phosphorus.^96^

#### Changing land use

If soil is heavily polluted, growing non-food crops and plants such as cotton, flax, broomcorn, grass, flowers and ornamental plants are the best remediation options.^10^ For instance, covering the heavily degraded alpine meadow in Qinghai-Tibetan with Elymus nutans significantly improved total concentrations of phosphorus, neutral phosphatase, urease, and catalase as well as upgraded microbial biomass carbon, nitrogen and phosphorus in soil.^97^ These non-food plants have economic value in addition to improving soil quality. Broom corn biomass could be used to make fiber and biogas, trees can be used for building materials, area greening, and seeds of castor oil plants can be used to make soap.^92^

#### Phytoremediation

Selected use of metal-accumulating plants for soil cleaning is an emerging technology which is low-cost and environmentally friendly.^10^ There are four main functions of this technology: phytoextraction, the use of metal accumulating plants to remove toxic metals from soil; phytovolatilization, evaporation of certain metals from aerial parts of the plant; phytostabilitzation, the use of plants to eliminate the bioavailability of toxic metals in soils; and rhizofiltration, the use of plant roots to remove toxic metals from polluted waters.^97^ For example, phytoextraction using non-irrigated rice cultivars grown for 2 years eliminated 883 g Cd ha^−1^, decreased the total soil Cd content by 38%, and minimized the grain Cd content by 47% in subsequently grown Japonica food rice.^98^ Selection of crop varieties with low heavy metal absorbability is very important for lowering heavy metals concentrations in the edible parts of plants to levels lower than food safety standards.^92^

#### Engineering techniques

This approach includes soil excavation, soil washing or burning to remediate metal contaminated soils. However, these techniques destroy the biotic components of soil and are technically difficult and implementation is economically unviable.^66^

### Impact of soil heavy metals

China feeds 22% of the world's population, but has only 7% of the world's arable land, and is hampered by soil quality problems mainly owing to the rapid rate of industrial development and urbanization.^13^,^99^ There is little doubt that soil contamination in China is becoming a threat to sustainable development.^9^ Soil pollution due to accumulations of heavy metals over natural background levels has led to environmental quality deterioration.^92^ About 10 million hectares of arable land has been polluted in China.^11^ Hence, high concentrations of heavy metals occur not only in soil, but also in food crops, particularly in southern China.^10^ A high proportion of rice grain exceeded the Cd limit in some areas of southern China, especially those areas impacted by mining and industrial activities.^100^ Furthermore, a recent survey of rice in the Xiangjiang river basin, Hunan province, found that 60% of sampled grain contained 0.2 mg Cd kg^−1^, while 11% contained more than 1.0 mg Cd kg^−1^.^101^ Another study in the Yangtze River region found that 37%, 16%, 60% and 70% of the sampled rice grain was contaminated with Cd, Hg, Pb and Cr, respectively.^102^ Residents living in contaminated areas who consume mainly locally produced grain and vegetables are particularly vulnerable to health risks.^10^ The entrance of heavy metals into humans through the food chain could lead to dangerous commutative health hazards. Due to the non-biodegradable nature of trace elements in the human digestion system, they have a long-term effect. High remediation costs have an adverse effect on China's economy. Heavy metal pollution has a great influence on ecological functions by harming the health of green plants and decreasing water quality.^29^ Soil pollution has multidimensional impacts on the economy (e.g. remediation costs), and on human health though the food chain, direct infiltration due to dermal contact, inhalation from air, as well as degrading ecological functions, threatening environmental sustainability.

#### Study limitations

The concentrations of heavy metals and the calculation of indices in this study were based on a review of the available literature from 2003 to 2015. Even though most of the reviewed papers were similar in terms of sample soil depth, there were disparities in the number of samples per unit area across the study locations. In addition, sample distribution was not spatially uniform and may not represent the comprehensive soil pollution situation in China. China is very large geographically, with diverse biophysical and socioeconomic settings. In addition, the magnitude of metal inputs into soil are changing over time due to rapid industrialization and urban development. Therefore, synthesis of the results from studies conducted at different times might underestimate pollution levels.

## Conclusions

The present study reviewed over 100 individual studies to determine the concentration, pollution level, sources, remedial actions and impacts of soil heavy metals in China. Although the natural level of heavy metals in soil is low in China, enhanced heavy metals concentrations is an increasing problem associated with rapid industrialization and agricultural intensification. Most concentrations of heavy metals were higher than their background values. There were variations in concentration and pollution levels among heavy metals in different places, signifying uneven distribution of anthropogenic influences. Spatially, soil pollution was more prevalent in urban than agricultural soils. At the national level, soil pollution was more widespread in southern China than in the north and west. Based on the integrated pollution index, about 53% of the provinces were in the category of moderate and heavy pollution levels (NIPI>2). As a result of soil pollution, green plants and agricultural grain products are increasingly polluted, creating public and ecological health risks. This has also impacted China's economy, such as remediation costs. The major sources of heavy metals in soil were waste water and gases, hazardous solid wastes and agricultural inputs. Natural sources such as soil parent materials and volcanic eruptions have low contributions, unlike anthropogenic sources. Different remedial actions have been taken to minimize soil heavy metals and to limit their mobility into the food chain. However, effective control of pollution sources and remediation of polluted soils have not yet been realized and are complicated by rapid industrialization, urbanization and agricultural intensification.
